# Being a Nursing Home Resident: A Challenge to One's Identity

**DOI:** 10.1155/2013/932381

**Published:** 2013-04-04

**Authors:** Maria Riedl, Franco Mantovan, Christa Them

**Affiliations:** ^1^Institute of Nursing Science, The Private University of Health Sciences, Medical Informatics and Technology (UMIT), 6060 Hall in Tirol, Austria; ^2^Nursing Department, University of Verona, 37129 Verona, Italy

## Abstract

Going into a nursing home can turn out to be a critical life experience if elderly people are afraid of losing their independence and identity after having moved into a nursing home. In order to find out what nursing home residents need in their first year after having moved into a nursing home to maintain their identity and self-determination, 20 problem-orientated interviews with residents of three nursing homes in the Austrian province of Salzburg were conducted and analysed based on content analysis according to Mayring. The participants of this study resist against having decisions taken away from them and fight for their independence and identity. In order to be able to cope with these strains, they need the help of family members, professionals, and identity-forming conversations in new social networks in the nursing home. The study participants draw enough strength from their faith in order to fight for their independence. They develop a new identity close to their previous identity by maintaining autonomy and mobility with a clear focus on the future.

## 1. Introduction

Currently, 16% of the entire European population are 65 years of age or older. From a demographic point of view, the European population is “twice as old” as the world population, whose proportion of senior citizens is 7%. According to forecasts, the proportion of senior citizens will reach 28% in 2050 [1].

Ageing from 75 years and onwards is characterized by critical changes and turning points, such as the death of a partner or a child, increasing health problems, the increasing need for care and possibly the move into an old peoples home or a nursing home [2].

In order to assess the current state of knowledge about the changes which the elderly experience in their first year after having moved into a nursing home, systematic literature research on “what changes do nursing home residents experience after their move into a nursing home?” was conducted [3]. Publications both in English and German, published between 1996 and 2010, were used. The following databases were utilised: Cinahl (Ebsco), Academic Search Elite (Ebsco), PsychInfo, Medline (PubMed), Embase, DIMDI, Gerolit, WISE, Cochrane, and Ageline. Keywords used are residential home/nursing home, home admission/moving into a nursing home, institutionalization, and burden.

People who move into a nursing home experience different types of changes which they feel to a greater or lesser degree is stressful. The change in social status, the impact on autonomy, the feeling of having no place to call home, the change in social contacts, and the reduction of habitual activities rank first in the presentation of the results and endanger the people's identity which they had before [4]. Moving into a nursing home is an individual experience [4]; the adaptation time frame is between 3 and 6 months [5, 6].

The experience in the nursing home is accompanied by a fight for autonomy and against having decisions made for them and actions imposed [7, 8]. Nursing home residents have experiences which they perceive as compulsive and degrading. They have to obey the staff in order to get on with them [9]. 

People living in a nursing home consider their self-determination at risk because they feel dependent on the daily nursing care process. Waiting for help and support is experienced as a feeling of powerlessness [10]. In addition, the nursing home residents experience a feeling of having no place to call home, as there are no private rooms available in nursing homes [11]. The boundaries between public and private spheres in the nursing home are seen as blurred in contrast to the clear boundaries that characterize the domestic home [12].

Social contacts are perceived differently after entering a nursing home than they were before. Nursing home residents still want to feel part of society outside the nursing home [10]. They also wish to remain in contact with family members and friends, thereby maintaining their social contacts from earlier times. This maintenance of earlier contacts demonstrates appreciation and respect for people living in a nursing home. It is sad to note, however, that contact between many nursing home residents and their families becomes rare after they move into a nursing home. The stated reason for this is a combination of lack of interest, a general avoidance of topics of disease, and homes for the elderly on the part of the family [7]. 

Lee [13] confirms that nursing home residents often advise their family members against visiting them because they do not want their family members to see life in a nursing home. The residents see their own dignity at risk because they are confronted daily with the increasing need for care, and support of the other residents [14]. In principle the dignity of nursing home residents appears to be based on social relationships [14], but it is actually the establishment of relationships among the nursing home residents which presents a particular challenge [7, 12, 13]. Nursing home residents require the support of staff when establishing interpersonal contacts, but their experience is that such support is inadequate. The nursing home residents experience the staff as people who are primarily responsible for maintaining order and discipline and spend their time dealing mainly with administrative tasks [13]. As a result the residents keep themselves to themselves and describe other residents as unfriendly and inactive. They make statements of feelings of dislike and repulsion towards their fellow residents [7]. 

According to Mateos et al. [15], nursing home residents complain that independence within the nursing home is given little attention. The motives of the elderly for staying active are insufficiently taken into account. Dependent behaviour is rewarded by the staff more than independent behaviour. The residents therefore hardly make use of their own capabilities because they feel that their abilities will be insufficiently recognised. Kane et al. [16] demonstrate that nursing home residents want to decide for themselves on that daily support which they receive in order to maintain their independence. 

According to Petzold [17], there is not enough time for fostering the competences of nursing home residents. Moreover, Petzold describes an overcompliance by nursing home residents. They do not protest, they display a high degree of subordination, they try to pander to all levels of staff in the home, and thereby they contribute to the progressive dismantling of their own competences. As a result the level of care needs increases, mental and psychosomatic disorders occur, nursing home residents socially withdraw, and they begin to lose their identity.

Therefore entry into a nursing home constitutes a momentous and significant event and turning point in the lives of the elderly. In fact, an ever shortening life span of residents after their entry into the nursing home is noticeable. 22% of residents die within the first six months of being in a nursing home [18].

### 1.1. Research Question

What is the experience of nursing home residents during their first year in the nursing home and how can they maintain independence and establish a social identity within the home?

In order to find the answer to this research question, a qualitative study [19] was conducted with nursing home residents in three nursing homes and homes for the elderly in the Austrian province of Salzburg.

## 2. Framework

To handle the concept of identity, the Identity Theory of Petzold [20–22] was chosen. Petzold [21] defines identity as the result of ego's synthesis performance in the processing of mutual (reciprocal) identifications from diverse social and cultural contexts (foreign attributions, foreign images), their emotional evaluation, the cognitive assessment and their connection with identifications (self-attributions, self-images). Petzold [20] also describes identity as the “answer to the questions “Who am I?” and “Who do I belong to?””

“Identity is what a human being gains by recognising himself through real perception and acting as the one he is (self-identification) and by being recognised by his fellow beings within his relevant environment as the one they see in him (identification from outside)” [22]. A human being's personality develops through social relationships. This means that identity develops via a process of social experiences, activities, stories, and dialogues with other people. Life situations and conversational partners change throughout the course of life, and therefore identity is to be seen as a constantly changing process. The formation of identity never ends [20].

Identity is subject to more or less considerable changes throughout the course of an individual's life. These changes include the alteration of living environment and bodily changes, particularly those due to age [22]. Elderly people like telling stories from former times again and again because they live in a world of memories from the past. Reminiscing and being in a state of thinking and reflecting means that contents of the long-term memory will emerge. These stories which stabilize identity are important as the elderly generate their sense of living from their reflection of the past [20].

Each story and all identity-forming elements of a story undergo “cerebral and mental processing,” which provokes a changeable identity in human beings. Therefore identity can be seen as a process that never ends and is constantly changing [21]. 

A human being's personality develops and grows in social relationships and includes five core areas (body; social relationships; work/performance/free time; material securities; values) from which a human being's identity is formed. 

**Figure 1:**
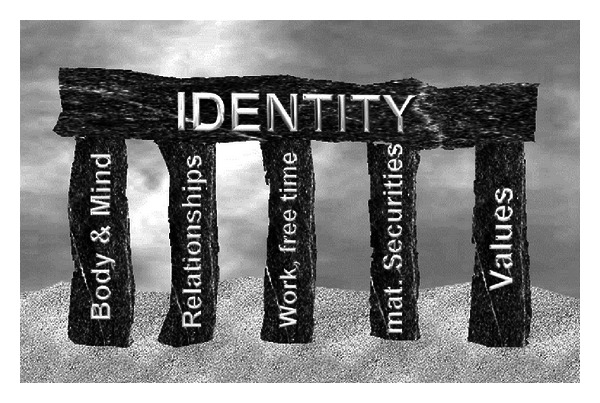
Five pillars of identity by Petzold (own drawing).

Identity-forming stories take place in the five essential identity areas that are described in the following. In these identity areas, one's own important individual experiences are shared with other people. These shared experiences of the people who tell the stories mutually influence their identities for the future. Metaphorically speaking, the five identity areas are called “pillars” (Figure 1) on which a human being's identity is based. The identity areas merge, and one area influences the other. The pillars are to be seen in a gender-specific manner [20–22].

According to Petzold [20], the level of strains placed on the identity of seniors is very high due to negative attributions and social deprivation. The ego no longer manages to perform synthesis. The ego weakens and is no longer able to stabilize a clear identity. If the social identity weakens at the same time, then a positive identification will be missing. 

**Figure 2:**
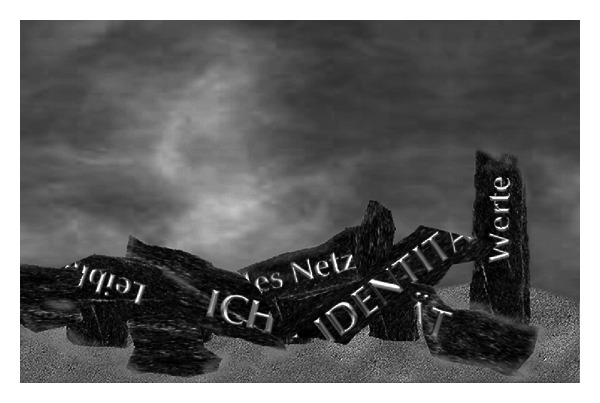
Broken pillars of identity (own drawing).

The identity is breaking down (Figure 2). The affected nursing home residents show a deterioration in performance, emotional exhaustion, crises of self-worth, and mental symptoms, such as aggressiveness and social withdrawal [20]. 

In order to gain information on how the elderly experience changes to their identity in the first year after having moved into a nursing home, a qualitative study was conducted in Austria.

## 3. Methodology

The study was comprised of residents (*n* = 20) from three nursing homes in the Austrian province of Salzburg. The criteria for inclusion in this sample were stays in a nursing home of less than one year, being more than 70 years old, physical and mental stability, not bed-ridden, cognitive ability to answer questions, competence to process information on the research subject, and no diagnosis of dementia. No sample size was determined in advance.

The access to the research field was gained by the first author, through contacts to the management of the nursing service of three nursing homes. 

The recommendations of Burns and Grove [23] with regards to the “informed consent” were implemented: study participants were informed about the aims of the study and the protection of personal data; the outcomes of the interviews were exclusively used for this study; the participants can revoke their consent at any time. The exposé was examined by an ethics committee; the committee confirmed the compliance with ethical principles. 

The data was collected through problem-orientated interviews according to Witzel [24]. The interviewee is seen as an expert on his own opinions and actions. The interviewer asks questions in order to generate narrations, alternating with questions in order to generate understanding. In order to facilitate the implementation of the interviews, short questionnaires, guidelines, a recording device, and postscripts are used.

The basis for the guidelines was due to the result of the literature research on the identity theory of Petzold [20–22, 25]. The semi-structured guidelines were divided into questions on their current life situation, review of the past, change of identity caused by moving away from home and into a nursing home, social contacts in the nursing home, and questions on expectations and wishes for the future. 

Before it was applied, the guidelines were tested in a test interview with a female nursing home resident who had fulfilled the inclusion criteria and were then modified accordingly. Some extensive questions were split into two or more questions. The result of the test interview was not included in the analysis. 

The interview was conducted with open questions in order to give the study participants space and time to talk about their experiences. All the study participants were asked all of the questions. However, the order of the questions was adapted to the progression of the interview.

The interviews were conducted by the first author of the study in the study participants' individual room. They were also conducted without a third party being present.

After each interview, the results were summarized then communicated, and discussed with the interviewee. The key aspects were documented in the interview record, and the results were approved by the study participants.

The interviews were analyzed following the transcription rules for computer-assisted analysis according to Kuckartz [26] in a slightly altered form; the study participants spoke a dialect, and therefore the texts of the interviews were polished. The records of the interviews with the study participants were then imported into the text memos of the MAXQDA program [26].

**Figure 3:**
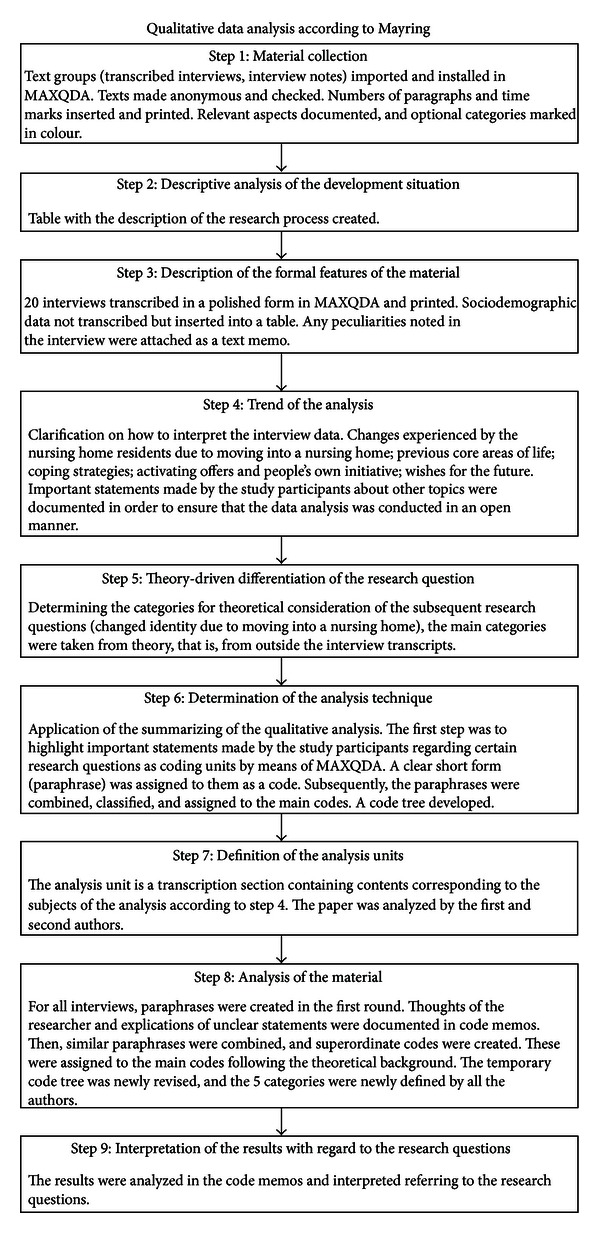
Process model of the qualitative data analysis (illustration compiled by the author).

The data was analyzed by means of the MAXQDA program [26] and was based on summarizing qualitative content analysis according to Mayring [27] following the nine-step process model (Figure 3) (material collection, descriptive analysis of the development situation, formal features of the material, trend of the analysis, theory-driven differentiation of the research question, determination of the analysis technique, definition of the analysis unit, analysis of the material, and interpretation of the results with regard to the research questions). The results were analyzed by the first and second authors of this paper [19] in order to gain a joint agreement on the final results.

The quality criteria of qualitative research [23] were met as follows:

- detailed processing of documentation—see Figure 3;
- safeguarding interpretations with arguments—see step 7;
- research process structured by rules of conduct—see step 6;
- closeness to the study participant—the interviews were conducted in the residents' bedrooms;
- communicative validation—after each interview, the results were summarized, communicated, and discussed with the participants; the results were then approved by the study participants.

### 3.1. Results of the Study

All interviews were conducted in July and August, in the year 2010. The study participants were aged between 71 and 93 (average = 82.35 years). The duration of the interviews was between 16 and 78 minutes (average = 35.5 minutes). The length of stay of the questioned nursing home residents was between 2 and 11 months (average = 7.2 months) at the time that the interview was conducted.

After a total of 20 interviews (15 women, 5 men) had been conducted, no new findings were evident from the authors' point of view with regard to the research question and aims. Hence, theoretical saturation had been reached. 

The core messages of the interviews were represented by means of a category system. Five core categories were derived from the data material.

#### 3.1.1. Category 1: Experienced Changes—Demands on Identity

When nursing home residents move into a nursing home, they have an emotionally stressful time, and they feel left alone and realise that their previous core areas of life are changing considerably. As they move into the nursing home, they find themselves in a state of shock, which is often accompanied with tears as it dawns on them that they are losing their home. *“Here, I realise that I do not have a home anymore” (B2, 18-18) “Now, that I have been institutionalized in the nursing home, everything is gone.” (B2, 105) “Here, you are in a waiting room for death.” (B3, 98) “This is a new phase, tears are falling.” (B2, 98) *[19].

The conditions of the fellow residents puts a strain on the study participants. “*When I had to pass people who I had known in younger days when they had been healthy and when I saw the condition they were now in, I was very shocked.” “One often thinks how pitiful people may become, first of all mentally.” (J1, 17-18)*. 

Due to having to go into a nursing home, identity-shaping core areas of life will change. The study participants feel exposed to a strongly reduced financial situation. *“One is a hardship case” (J8, 49). “My friend said: Do not move into a nursing home. They will take all your money there.” (J2, 75)* [19].

The loss of previous work gives the nursing home residents the feeling of being nothing and a nobody. “*It's a new situation if one moves into a nursing home. At home, I used to cook for my children and grandchildren. There I had a task to fulfill.” (S1, 90) “Moreover, I still used to sew and knit everything for the kids myself.” (J1, 26). “But here, nobody mentions that anymore.” (J8, 15)* [19].

The study participants, who experienced the process of moving into a nursing home positively, highlight the advantages of living in a nursing home.* “Here, I have someone to talk to. At home, I used to be alone because my daughter works” (J9, 33-34)* [19].

#### 3.1.2. Category 2: Coping with Changes

The process of moving from one's own flat into a nursing home is handled very differently by each study participant. In the adaptation phase, nursing home residents talk to others about their feelings, such as sorrow and home sickness. Some study participants cope throughout the arrival period with comforting prayers. “*I draw strength from praying, I consult with Mother Mary” (J9, 95).*

Visits to the cemetery are described as very comforting by several of the study participants and help them cope better with what they experience.* “I often visit the grave of my parents-in-law. I take care of the flowers and talk about my worries at the grave side” (J8, 95) *[19]. In the interviews, the nursing home residents repeatedly pointed out that they found comfort in faith. *“Yes, I am indeed a believer. I pray often. Yes, now I have time for it. Yes, thus I can cope better with it. Faith gives me support. Yes, you can take your faith with you to the nursing home” (S2, 142, 162)* [19].

Some study participants overcome their grief by talking about it to others. *“I also have bad times, indeed. Last time I told my GP that I have a feeling like homesickness. It is a feeling of longing for something. You are full of thoughts. And this makes you sad. I also talk about it with my children. They acknowledge that they had a loving childhood home. This makes me feel relieved.” (J1, 33) “This is not really my home. When they pick me up and take me back, I always think to myself: Here, I am not at home.” (J6, 38). “However, I understand that this is the only solution. At the age of almost 90 one must be happy about this situation.” (J6, 40). “Of course, moving into a nursing home was a shock for a bit. One has to chat with the other nursing home residents, then it is actually quite ok.” (S1, 44-46)*.

One female study participant regularly talks about her grief to her teddy bear which she took with her from home because she does not want to be a strain on her children. *“Yes, and the teddy… I always have him with me. I need him to sleep. I took him with me from home. I talk to him, I tell him everything. He sleeps in my bed. There, I talk to him.” (S1, 135).*

Some of the residents questioned are still struggling to overcome their grief. *“I will have to put up with it. At the moment, it's better. I was crying a lot. I talked about it with my doctor, and then he prescribed some medication for my nerves. It has got better” (J7, 52-54). “To be honest, if it was possible, I would love to die immediately” (B7, 102).*

Some nursing home resident's cope with the situation positively by highlighting the advantages of living in a nursing home; for example, help is available all around the clock.* “I prefer living in a nursing home.Yes, because here I know all the nurses. I just have to push the button and a nurse comes at once” (B5, 41).*

#### 3.1.3. Category 3: Maintenance of Autonomy through Mobility

According to Kruse and Wahl (2010) the concept of health in old age focuses on the ability to lead an autonomous and independent life despite having an illness. In this context, the individually chosen and adopted strategies to find meaningful activities are important [28]. 

The study participants, more often than not, downplay the offers made by the nurses to support them in the handling of their physical deficits. They refuse to believe in their shortcomings and say that they can do everything themselves, apart from cleaning and cooking. It is to be noted that the participants of this study ensure that they themselves stay mobile and independent by taking walks to the cemetery and to their former living areas, as well as by taking trips and train and bus excursions. In particular, it is highlighted how important it is to have the opportunity to leave the nursing home without any nursing home employees at any given time. This possibility gives the nursing home residents a feeling of freedom. *“We, the women of the nursing home, go for a walk almost every day. Yes, once a week I visit my cat at my old house. Then I pet her and say: “My beloved Cindy.” What a pity. But she wants for nothing.” (J4, 84, 28) “I do not have any relations in the nursing home. No, I do not have any. I often go for a walk, go shopping or stroll along the river. I know some of the people who walk there. That is all I have.” (S4, 162). “Sometimes I go to friends by bus because I have a senior citizen's ticket. In the evening I go back again to the nursing home” (J2, 57) *[19]*.*

For one study participant it is of particular importance to have a key and thus the possibility to leave the nursing home at any time.* “Mostly, I do not attend the in-house dinner. At dinner time I drive around the village center in my wheelchair with some friends of mine. I tell a member of staff when I leave the nursing home. This is not a problem. Sometimes I am not back before midnight. I have a key. So, I can come and go whenever I want. That's great. Because the staff do not have to give a key to the residents.” (B1, 6).*

#### 3.1.4. Category 4: Establishment of a New Identity by Creating a “New” Normality

In the present study, the participants compared normality in the nursing home with their life situation at home, before they moved to the nursing home. 

Elderly people who are moving into a nursing home try to keep as many of their habits as possible from the good old days. The new normality is adapted as closely as possible to their previous life. When this new normality has been accepted, this new place of living can then be seen as home. Nursing home residents try to reestablish “normality” by arranging their new room accordingly, for example, using the furniture from their previous home. These objects from the good old times, when the study participants were younger, shape their identity and provoke positive memories. *“My GP said: “your place in the nursing home is like it used to be in your old home”. At home, my photos were also placed right above the sitting corner, just like they are here in the nursing home” (J1, 23)* [19].

The study participants wish to partake in activities in the nursing home that are comparable to their previous hobbies and roles in life. The activities serve to keep the residents occupied, but also to stabilise their identity. Activities give the study participants a good feeling of being useful. *“I have my hoover with me and hoover my room. The children take their shoes off when they come in. I make the bed myself. Yes, that's quite nice. I am satisfied” (B2, 65, 67) “I live with the flowers. I have already done something, have a look. Carnations, I look after them. I water them and cut them” (J8, 44)*.

In the interviews, the nursing home residents who were questioned repeatedly pointed out that they found comfort in faith. *“Yes, I am indeed a believer. I pray often. Yes, now I have time for it. Yes, thus I can better cope with it. Faith gives me support” (S2, 142, 162)* [19].

The study participants partly reject social activities that are offered to them by the employees of the nursing homes. They complain about conflicts amongst the residents and therefore keep out of one anothers' way. *“Yes, there is always one person who is jealous of everyone. I get the pullovers from my sister, they envy me, and also my bedroom. I simply avoid these people” (S4, 78) *[19]. 

#### 3.1.5. Category 5: Wishes and Expectations for the Future

The new nursing home residents feel healthy and wish that their physical state will not change. *“I have no expectations anymore. I want to stay healthy and then die quickly” (J1, 59)* [19].

Some study participants express fear of becoming a person in need of care. *“I have no expectations anymore. The principal thing is not to become a nursing case. I do not want to become an invalid as some of the other residents. I do not want to lose my mind. In this case, I would rather die” (J1, 59).*

The elderly are realistic when it comes to the end of life. *“It is sad. One should not become that old. If it was possible, I would like to die, yes.” (B7, 130) “In the past, I was very afraid of death. And now I think it is pleasant. I sometimes even look forward to dying” (B9, 59).*

Some study participants express the longing for death; they actually wish to die. *“Dying would be alright with me at any hour before I have to suffer, as I can see so many here do. Then I think to myself that I want to fade away now. One is always confronted with sorrow, with so many images of bad conditions” (B2, 53-54)* [19].

The participants of this study are glad to have arranged everything in the case of their death. *“Yes, I wish to have a nice place in my husband's and son's grave. After my son died, we bought a grave in a place where a tree had been before. We selected this place. There is a place left for me which is as beautiful” (J5, 145)* [19].

## 4. Discussion

According to Riedl et al. [3], the elderly's experience of moving into a nursing home is unique to each and a critical moment in their life. Despite preparation for this critical moment, the move into a nursing home causes change in social status, impact on autonomy, the feeling of having no place to call home, change in social contacts, and the reduction of habitual activities all of which up until this point has formed this persons' identity. In the adaptation phase during the first half year in the nursing home, many of the residents feel over-challenged and unable to cope with the new situation. The other residents are perceived as a strain, and the nursing staff are seen as the ones responsible for upholding discipline and order with little time for supporting new residents. The nursing home residents miss a private space which should be their new home. In this period, the identity of the elderly is endangered and threatens to break down [3]. 

For the participants of this qualitative study from Austria, “the experienced changes” were a great challenge to the identity's lived up until this point. On the one hand several pillars of identity were weakened by emotional pressures such as homesickness, perceived changes in the main focus of their lives, and the changed financial situation. On the other hand, the participants of this study showed that they still possessed enough emotional resources to view the move into the nursing home as reasonable after a few months. They carefully select residents as conversation partners who do not cause emotional strain through their need for care. The willingness to talk to others instead of withdrawing helps establish their future identity [21]. Nursing home residents who's condition is perceived as being emotionally stressful either by confusion or a high level of physical care will be avoided as conversation partners. According to Petzold, identity is the “answer to the questions “Who am I?” and “Who do I belong to?”” [25] and thus can be seen as the reason for the rejection of communication with confused residents. 

Similarly as in the study of Hanisch-Berndt and Göritz [29], nursing home residents choose a strategy of rejection in order to maintain a self-image that is as positive as possible. The participants of this study confirmed the importance of a positive self-image by describing the physical support given by the nursing staff as completely insignificant. In the interviews, support by the nursing staff was only mentioned in the context of household activities, such as washing clothes and cleaning the floor. In doing so, the participants of this study confirm Pleschberger's work [14] which supports the view that nursing home residents see their dignity in danger when confronted by an increasing care requirement for themselves. Financial concerns correspond with the studies of Kane et al. [16]. The nursing home residents strive to have money of their own. This brings a degree of independence, creates a desired freedom, and strengthens the pillar “material security” [20].

As stated in the study by Heliker and Scholler-Jaquish, the study participants seek out people with whom they can discuss and share their problems with in order to “cope with the experienced changes positively” [11]. According to Petzold [25], genuine personal encounters are possible when the nursing home residents exchange their experiences with each other. New social spheres develop through common experiences, and the pillar of “social relationships” is stabilized. Thus, the first steps towards settling into the new home environment have been taken. In addition sharing one's own stories with the nursing staff is important in order to get to know one another. According to Pleschberger [14], nursing home residents dignify themselves not only by their behaviour with other residents and nursing staff but also through what they have achieved during their lifetime. Moreover, the study participants can work on maintaining their own mobility and autonomy by not requesting more help than is necessary from the nursing staff and thus considerably strengthen the pillar of “physical well being.”

Religious rituals, such as pray and regular visits to the graves of deceased family members, help the study participants cope with their entry into the nursing home. People will be identified in their environment and chosen as new friends, if they show a similar attitude to that which they exhibited prior to entering the home. The “pillar of values” is strengthened. The study participants ensure for themselves, through their means of coping that their changed identity remains stable and that communication with social support networks continues.

Striving for “maintenance of autonomy through mobility” is constantly highlighted throughout this study. As also stated in the literature of Kruse and Wahl [28], the concept of health in old age focuses on the ability to lead an autonomous and independent life despite any illnesses. In this context, the individually chosen and adopted strategies to find meaningful activities are important. The participants of this study say that they do not suffer from physical deficits. They do this in order to avoid being at the risk of being someone who is regarded as in need of support and of having everyday life in the nursing home organised for them by the nursing staff. The freedom to decide when you leave the nursing home and with whom ties in with the importance of maintaining the previous identity. The institution's impenetrable boundaries as described by Gamliel [30] are thus removed. 

The physical care as mentioned by Koppitz [10] is accepted at both the beginning and end of the day, but during the day the study participants strive for autonomy. Through their own initiative, they are active and plan their everyday life themselves. In doing so, the study participants have the feeling of personal integrity, self-assertiveness and have the personal freedom in which to decide how to spend their time. Former preferences may even be reactivated. Thereby the participants of this study seem largely satisfied with their life in the nursing home. As stated by Mateos et al., they stay active in order to be able to take part in the activities which are meaningful to them [15]. The nursing staff at the Austrian nursing homes, who was chosen for this sample study, does provide the required social support [8]. This is done by not preventing the study participants from performing their planned activities. 

If mobility and autonomy are preserved to a sufficient extent, the study participants start the “development of a new identity and the establishment of a “new” normality.” Memories are kept alive in their room in the nursing home through the furniture and objects which they brought with them and thus inspires them to tell stories from earlier times. Photos, crockery, items of clothing, and so forth strengthen their previous identity. Activities, which can no longer be performed due to being in a nursing home, such as running the household, are upheld through stories, giving these memories a positive tinge. As in Petzold's study [25], the participants also generate their sense of life through reflecting the past. 

The study participants tell their stories and exchange their experiences every day. As confirmed by Breyl [7], this is the way in which they tie in their previous life outside of the nursing home. For example, this is how female study participants who themselves have raised children can continue their role as a mother through their stories. As described by Heliker and Scholler-Jaquish [11], stories that are told which people have in common, manifest interest. Just as the people questioned in this study make new friends in the nursing home through the stories they share with each other. The study participants take several core areas of their lives into the nursing home with them, such as religious rituals and knowledge from their previous everyday life.

They can continue to pursue their hobbies in the nursing home or aspire to taking up a new hobby. One study participant, for example, enjoys doing handicrafts and confesses to having never had time for handicrafts before. As proved by Lee [13], the participants of this study also make an effort to reestablish their new identity and their “new” normality as closely as possible to their previous life. The study participants manage to define clear boundaries, and according to Hauge and Heggen [12] clear boundaries characterize life at home. After they have succeeded in stabilizing the pillars of their identity, even though they have moved into a nursing home, the study participants live their new identity as nursing home residents in a largely self-confident and satisfied manner after a relatively short period of time. 

This fact underlines changeable identity as described by Petzold [22], which according to the results of this study can also be coped positively by the elderly (average = 82.35 years). 

The “wishes and expectations” of the study participants are congruent with the remarks of Mahs [31]. The participants of this study wish their families to be well and to stay healthy themselves. They rate their health status as good and hope for many more healthy years. However, they are afraid of becoming an invalid beforehand. 

The study participants are not afraid of dying. They know that death is a part of life. They have made all the preparations in the case of their death. Kruse [32] confirms this fact by stating that the preparation for death is a borderline situation which makes people grow and develop. According to Kruse, elderly people have the feeling that death occurs at the right point of time in old age. 

Similar to the study of Mahs [31], the people questioned in this study also believe in resurrection. According to the results of this study, they find comfort and hope in faith and draw strength from it, as Petzold described, for their life in the nursing home [22]. From the stated wishes and expectations, the conclusion can be drawn that most of the formulated wishes do refer to their own death, but that the people questioned are satisfied with their situation in the nursing home, and that they have managed to establish a “new” identity which gives them hope for a good and positive future.

## 5. Conclusions

On the one hand, the results of this study show that moving into a nursing home is a critical life experience, but on the other hand, elderly people manage to cope with this move and can create an identity for their future. In order to be able to cope with the demand on their identity, they need identity-forming conversations in new social networks in the nursing home as well as the support from their family members and professional helpers. 

**Figure 4:**
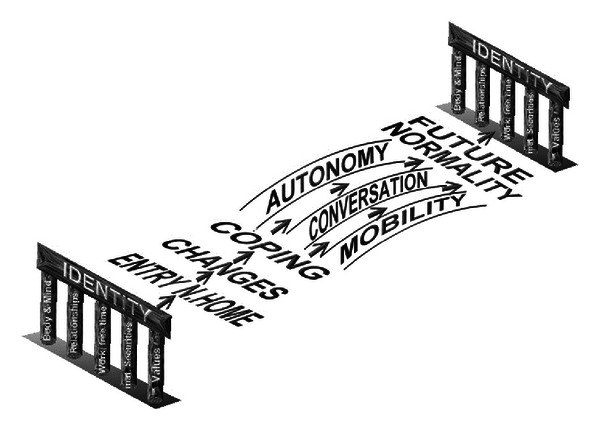
Entry into a nursing home and future identity (own drawing).

Figure 4 is an attempt to represent the path to a changed identity as a nursing home resident. The identity of the elderly is based on their entire previous life. Moving into a nursing home destabilizes the identity that had been lived up to that point in time. The first challenge which the affected people face is coping with the psychophysical and social changes [3]. 

Every change that is accepted leads to an adaptation to the new situation. Another task in the adaptation process is to maintain autonomy and mobility in order to be able to take part in decision making and to experience a positive self-image. In doing so, progressive degeneration of competences in the elderly people can be counteracted [19, 32].

Identity is also changeable in old age and can live up to the demands of moving into a nursing home. The new identity is formed on a mutual basis through intersubjective interaction with different people in social networks. A common bond develops by telling identity-forming stories. If this happens, nursing home residents can build a normality close to their previous normality. A stable, changed identity (Figure 4) develops. Nursing home residents take part in the decision making processes regarding life in the nursing home and thereby remain future orientated.

## 6. Implications for Practice and Research

From the results of this study, recommendations on how to act when somebody moves into a nursing home and on the adaptation phase of the elderly within the nursing home can be deduced. Elderly residents need to be supported by their participation in decision making when selecting a nursing home, by determining the time of the entry into the nursing home, by the maintenance of a support network in and outside the nursing home, by involvement in financial matters, and by the maintenance of mobility and autonomy. A narrative climate should be established in order to make identity-forming story telling possible.

In order to be able to give nursing staff and those with political responsibility reliable recommendations for action, further quantitative, and qualitative studies are required. The results of this study provide ideas for those areas in which further measures can be developed.
